# Supplementary material for: Mutational profile of the regenerative process and de novo genome assembly of the planarian Schmidtea polychroa
Source: Nucleic Acids Res. 2024 Jan 5;52(4):1779–92. doi: 10.1093/nar/gkad1250 (PMC10899757; doi:10.1093/nar/gkad1250)
Supplement: gkad1250_Supplemental_Files [file gkad1250_supplemental_files.zip › Supplementary figures.pdf]

Figure S1

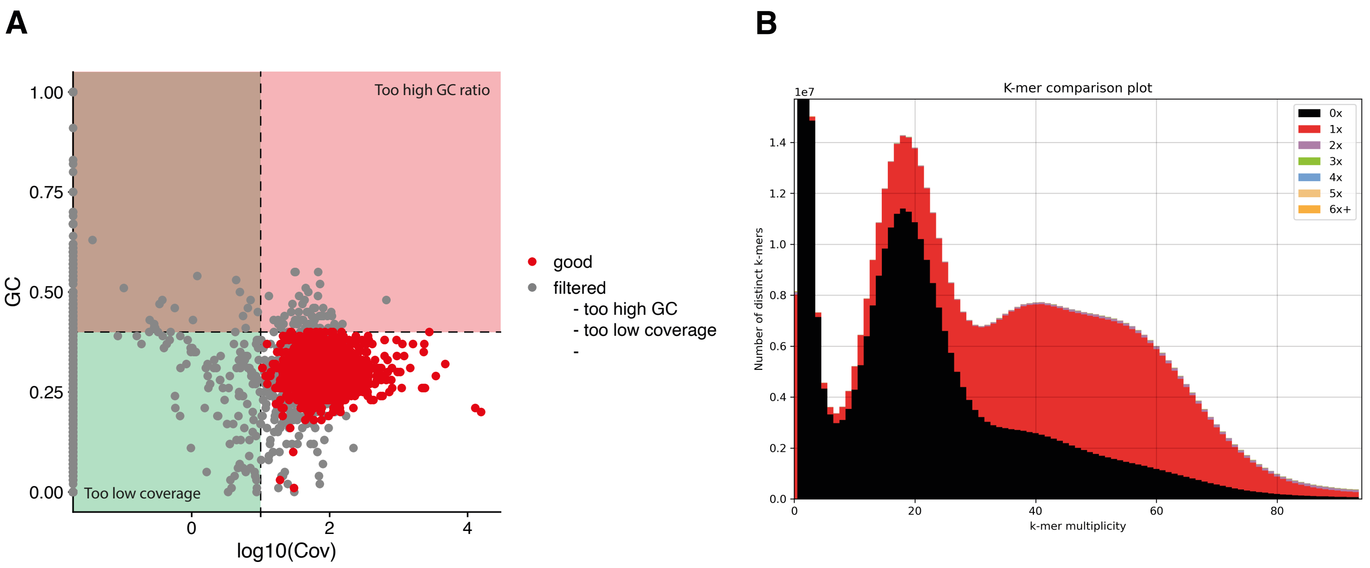

Figure S1. Genome assembly quality control. A) Scatter plot of GC ratios (Y axis) and Illumina coverages (X axis) of purged scaffolds. Grey points refer to scaffolds that were filtered either because they had GC ratios over 40% (pink region) or because they had coverages less than 10x (green region). Only contigs marked by the red markers were included in the final bm_Spol_g1 assembly. B) KAT profile of the final assembly. The three peaks suggest that the S. polychroa genome is triploid, the relatively large black region belongs to the genomic regions missing from the assembly, and the virtual lack of k-mers present more than once suggest that the assembly is appropriately purged.

Figure S2

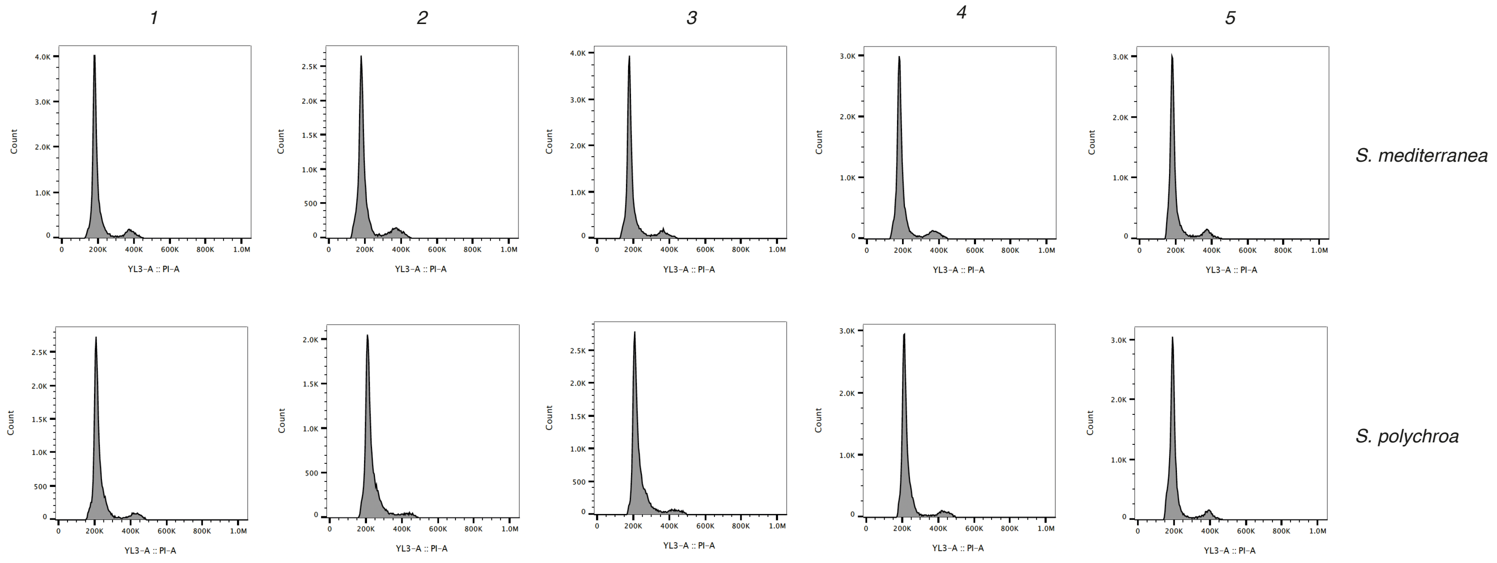

Figure S2. FACS profiles of all 5-5 S. polychroa and S. mediterranea samples for the genome size determination experiment.

Figure S3

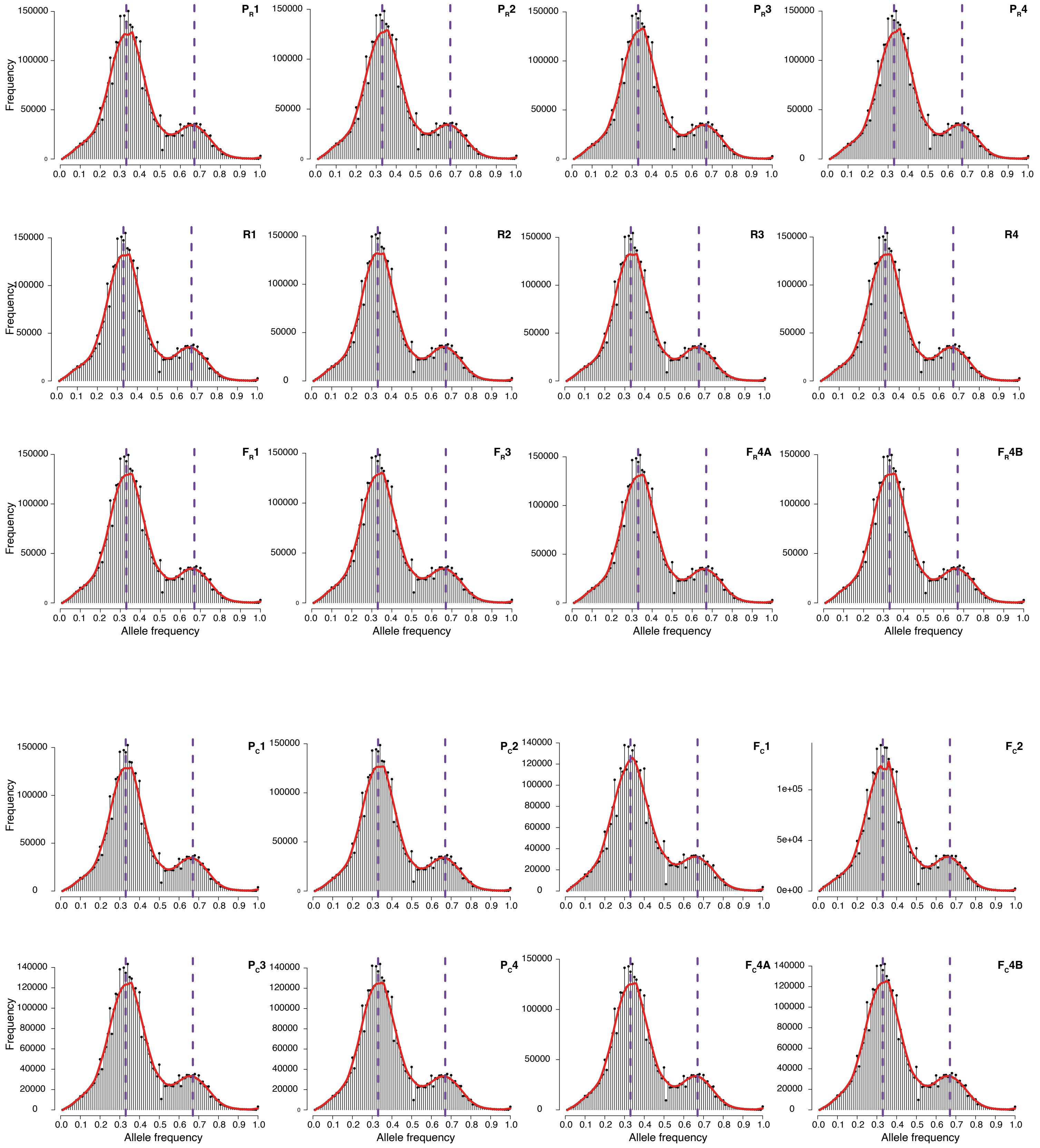

Figure S3. Allele frequency distributions of the analysed animals. The red line marks a lowess approximation of the observed counts.

Figure S4

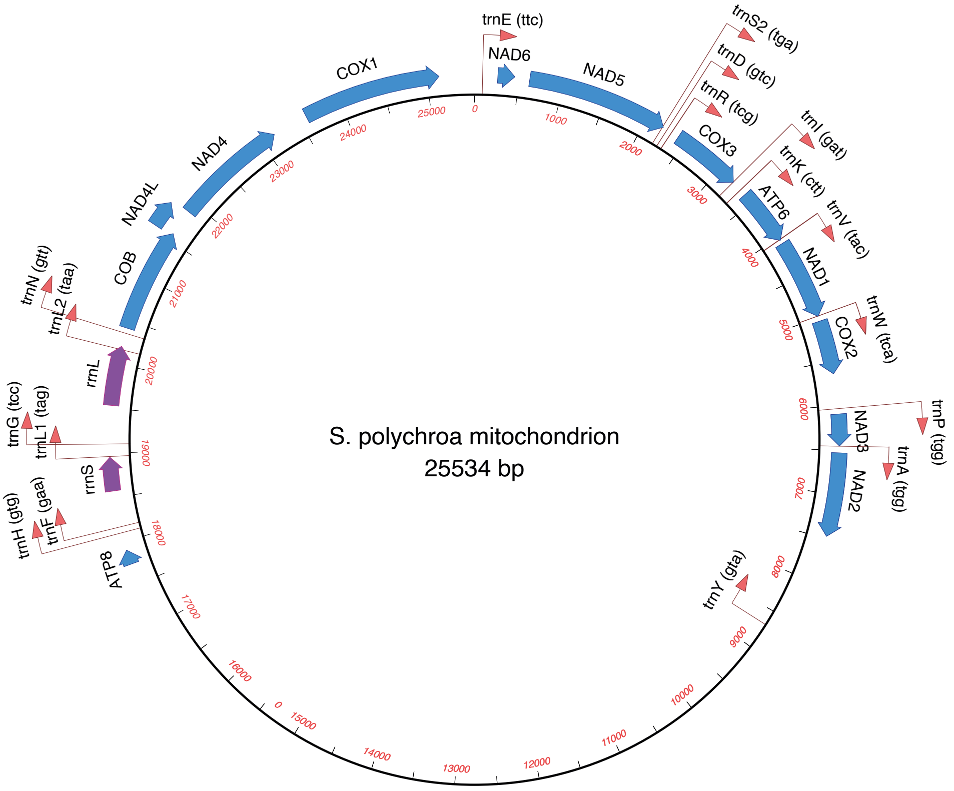

Figure S4. Annotated map of the mitochondrial genome of S. polychroa. Blue arrows: protein coding genes; Purple arrows: ribosomal RNA genes; red markers: tRNA genes.

Figure S5

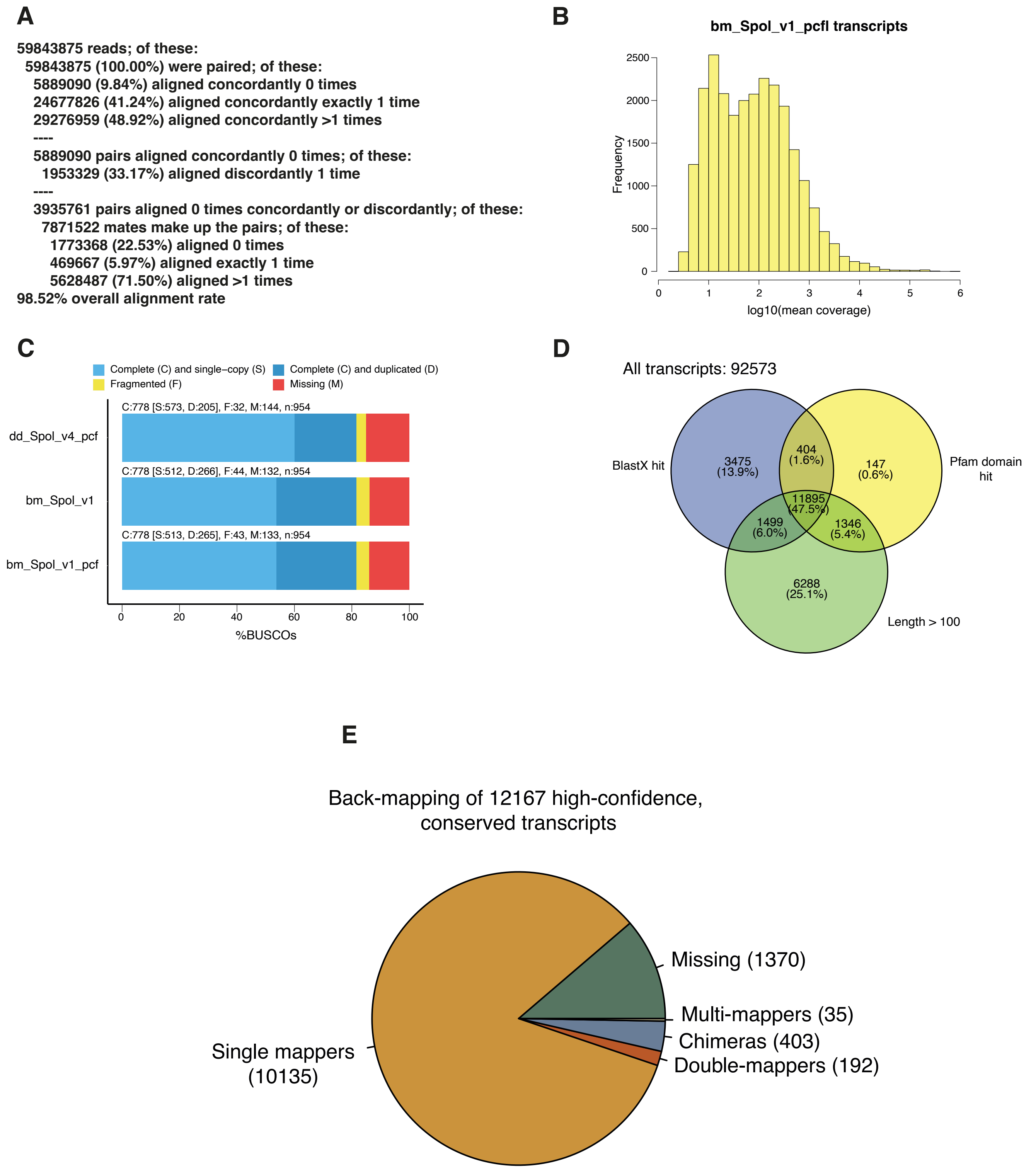

Figure S5. A) Bowtie2 alignment statistics after mapping the RNA-seq reads used for the assembly onto the bm_Spol_tr1transcriptome assembly. B) Histogram of mean coverages of the RNA-seq back-mapping. C) BUSCO outputs of the raw and the probable protein-coding fraction (PCF) filtered bm_Spol_tr1transcriptomes, compared to the dd_Spol_v4_pcf transcriptome downloaded from PlanMine. D) Venn diagram of transcripts affected by the three filtering factors (BLASTx hit, Pfam domain hit, protein length > 100 amino acids) for the PCF set generation. E) Back-mapping results of PCFL transcripts from the bm_Spol_tr1 transcriptome that could be matched with a dd_Spol_v4 and a SMEST.1 transcript. The categories are identical to the main classes on Fig 3A.

Figure S6

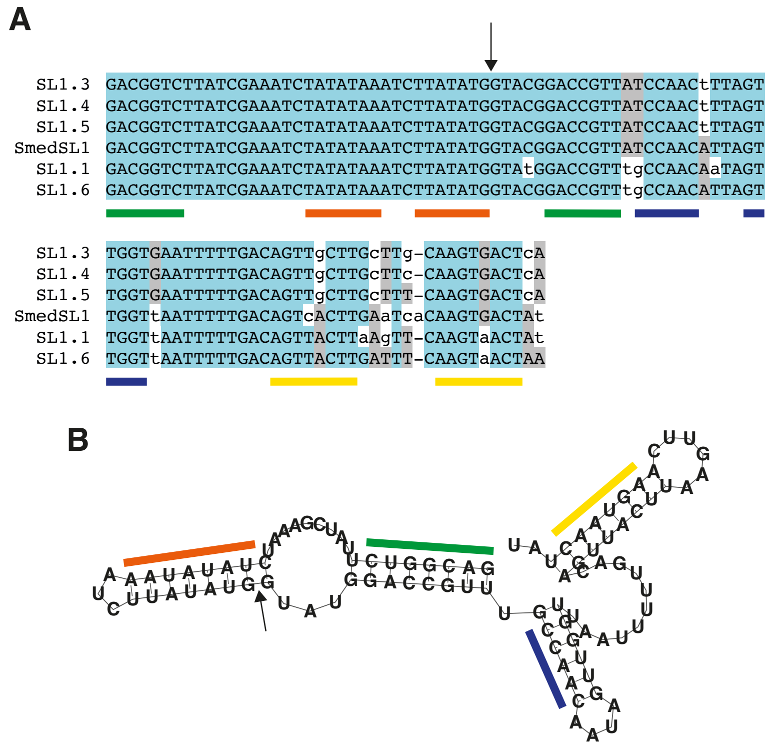

Figure S6. Spliced-leader sequence in S. polychroa. A) Alignment of the spliced-leader RNA genes found in S. polychroa and the SL1 gene sequence from S. mediterranea. The colored segments mark stem regions. B) Secondary structure of the S. polychroa spliced-leader RNA. The colored segments are identical to the regions on A). The splice-site is marked wirt a black arrow.

Figure S7

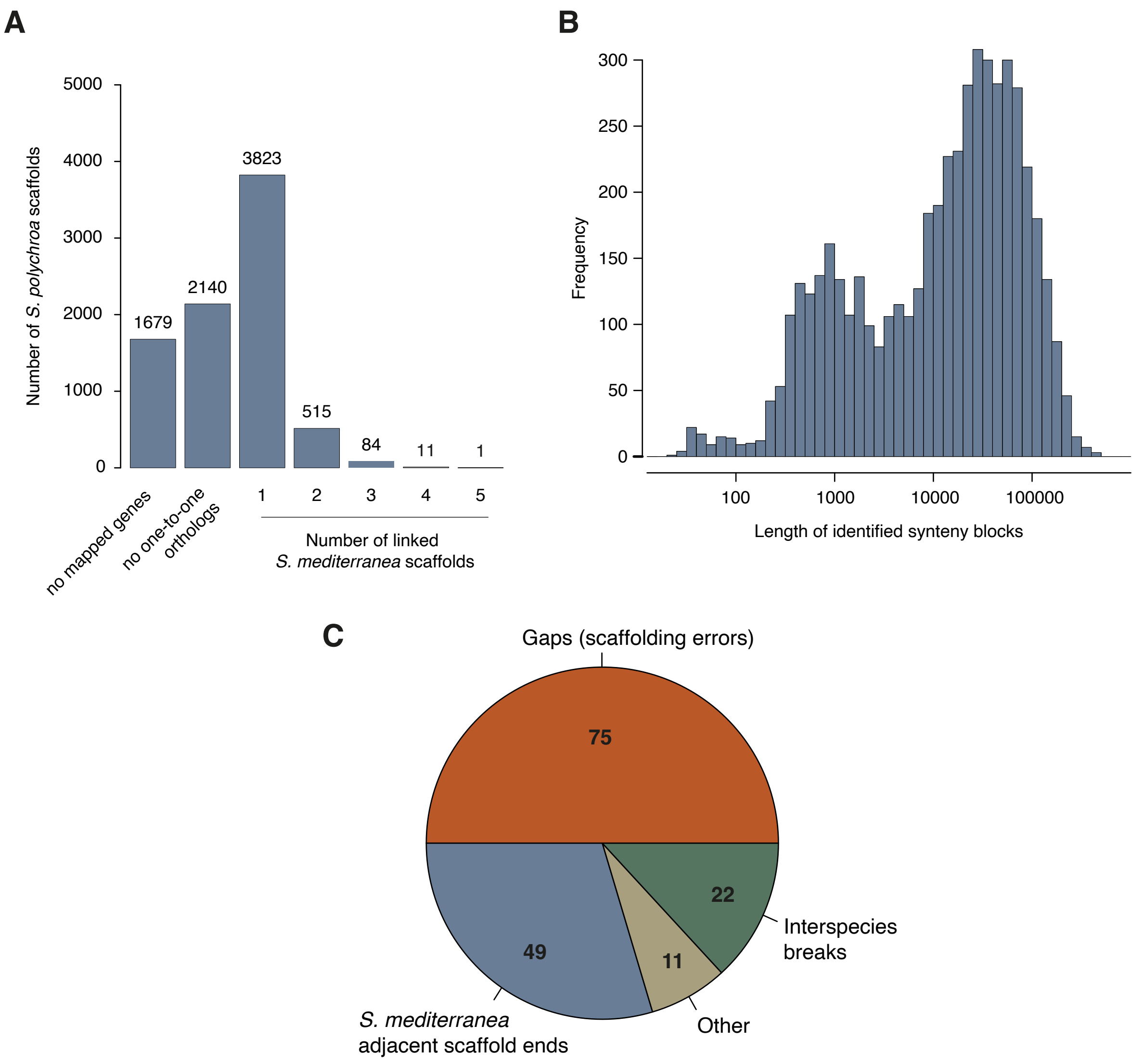

Figure S7. A) Counts of S. mediterranea scaffolds corresponding to each S. polychroa scaffold, determined using one-to-one homologous gene pairs from PlanMine. B) Distribution of synteny block lengths, defined as the start of the first and the end of the last gene in a block. C) Detailed analysis of regions corresponding to observed rearrangements between the two Schmidtea species. Only breakpoints with at least two genes on both sides in the S. polychroa assembly were considered, and misassemblies were defined as breakpoints with zero coverage in both Illumina and PacBio alignments.

Figure S8

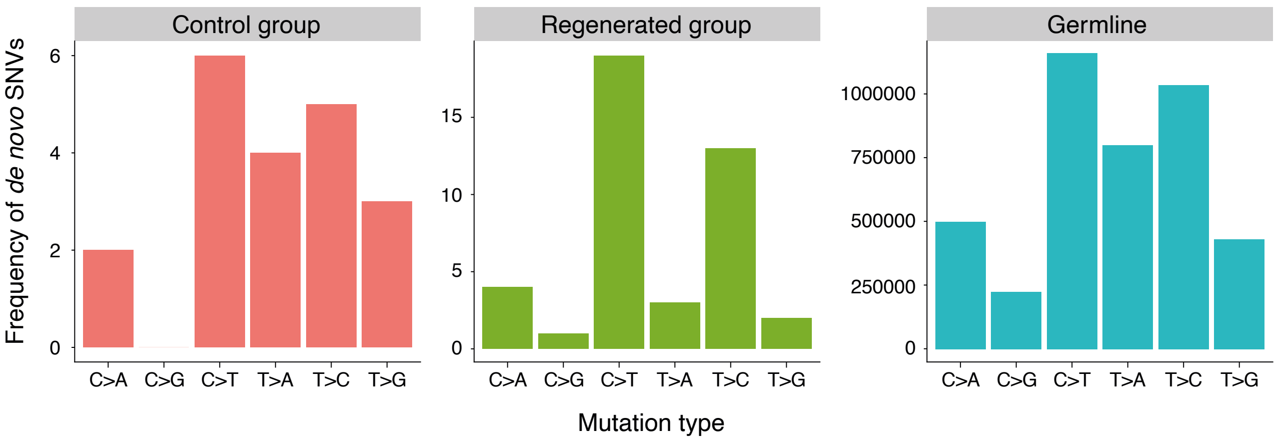

Figure S8. Substitution spectra of de novo mutations in the control and regenerated groups, and of germline mutations present in all animals.
